# Supplementary material for: Morphological and Transcriptome Analysis of the Near-Threatened Orchid Habenaria radiata with Petals Shaped Like a Flying White Bird
Source: Plants (Basel). 2025 Jan 28;14(3):393. doi: 10.3390/plants14030393 (PMC11820888; doi:10.3390/plants14030393)
Supplement: Supplementary file 1 [file plants-14-00393-s001.zip › plants-3419571-supplementary.pdf]

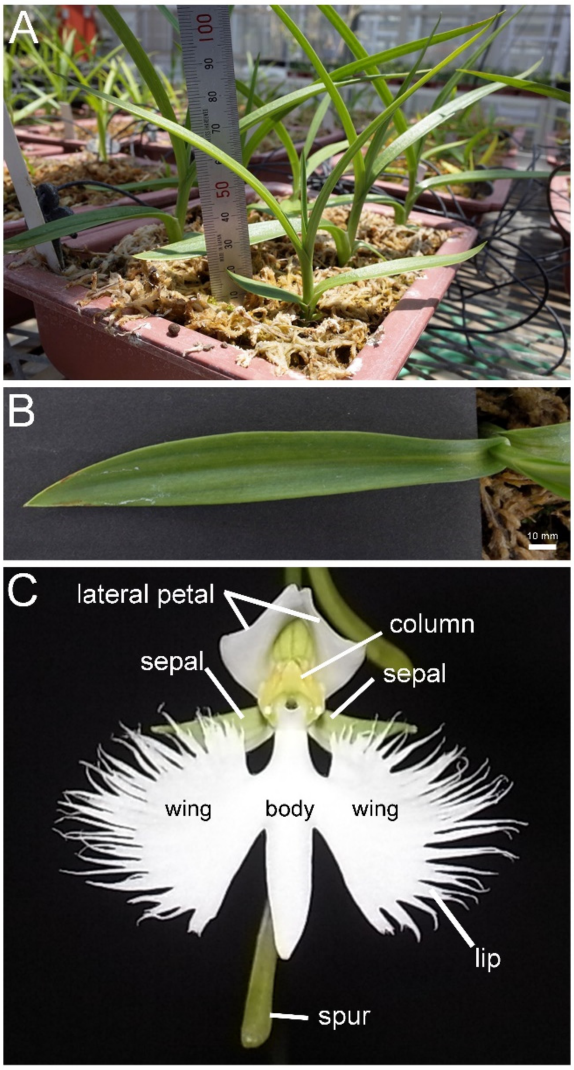

Figure S1. Morphology of *Habenaria radiata*. (A) Formation of leaves during the vegetative phase. (B) Simple leaf. Scale bar = 10 mm. (C) Flower consisting of three green sepals, two lateral petals, one lip (labellum), and one column. A spur is formed at the base of the lip, storing liquid nectar at its tip. The lip consists of a central body part and two lateral wings.

Table S1. Gene ontology analysis of 6,127 genes expressed in floral buds with 3, 4, and 5 mm in length

**GO biological process complete**	**Fold enrichment**	**P-value**
mitotic DNA damage checkpoint signaling (GO:0044773)	4.12	6.68E-03
DNA damage checkpoint signaling (GO:0000077)	4	3.39E-05
mitotic DNA integrity checkpoint signaling (GO:0044774)	4	3.39E-05
double-strand break repair via nonhomologous end joining (GO:0006303)	3.88	2.13E-03
negative regulation of cell cycle G2/M phase transition (GO:1902750)	3.88	2.13E-03
DNA-templated DNA replication maintenance of fidelity (GO:0045005)	3.83	8.33E-03
mitotic G2/M transition checkpoint (GO:0044818)	3.83	8.33E-03
negative regulation of G2/M transition of mitotic cell cycle (GO:0010972)	3.83	8.33E-03
double-strand break repair via break-induced replication (GO:0000727)	3.77	3.21E-02
signal transduction in response to DNA damage (GO:0042770)	3.77	1.61E-04
DNA integrity checkpoint signaling (GO:0031570)	3.69	2.47E-07
DNA replication initiation (GO:0006270)	3.62	3.37E-06
chromosome condensation (GO:0030261)	3.58	6.09E-04
mitotic cell cycle checkpoint signaling (GO:0007093)	3.51	2.42E-07
negative regulation of cell cycle phase transition (GO:1901988)	3.48	1.02E-10
negative regulation of mitotic cell cycle phase transition (GO:1901991)	3.48	1.16E-05
cell cycle checkpoint signaling (GO:0000075)	3.45	3.56E-10
DNA replication checkpoint signaling (GO:0000076)	3.4	2.71E-02
mismatch repair (GO:0006298)	3.34	6.62E-03
negative regulation of mitotic cell cycle (GO:0045930)	3.33	6.59E-07
regulation of meristem structural organization (GO:0009934)	3.29	1.62E-03
regulation of G2/M transition of mitotic cell cycle (GO:0010389)	3.19	3.18E-04
regulation of cell cycle G2/M phase transition (GO:1902749)	3.17	7.79E-05
DNA duplex unwinding (GO:0032508)	3.07	7.84E-04
DNA geometric change (GO:0032392)	3.07	7.84E-04
negative regulation of cell cycle process (GO:0010948)	3.05	2.60E-09
regulation of cell cycle phase transition (GO:1901987)	3	5.72E-12

regulation of chromosome organization (GO:0033044)	2.98	2.64E-05
DNA conformation change (GO:0071103)	2.9	5.81E-05
floral organ formation (GO:0048449)	2.9	2.22E-02
response to ionizing radiation (GO:0010212)	2.9	2.22E-02
negative regulation of cell cycle (GO:0045786)	2.89	2.93E-08
leaf vascular tissue pattern formation (GO:0010305)	2.88	9.56E-04
regulation of mitotic cell cycle phase transition (GO:1901990)	2.87	1.89E-06
DNA-templated DNA replication (GO:0006261)	2.82	1.67E-15
mitotic sister chromatid segregation (GO:0000070)	2.82	4.92E-04
mitotic nuclear division (GO:0140014)	2.79	1.56E-05
microtubule-based movement (GO:0007018)	2.78	5.03E-07
sister chromatid segregation (GO:0000819)	2.76	2.47E-04
cell fate specification (GO:0001708)	2.74	5.59E-03
DNA replication (GO:0006260)	2.72	1.63E-16
floral organ morphogenesis (GO:0048444)	2.72	2.19E-05
regulation of cell cycle process (GO:0010564)	2.72	1.38E-18
nuclear chromosome segregation (GO:0098813)	2.71	5.30E-10
chromosome segregation (GO:0007059)	2.69	2.22E-16
embryonic meristem development (GO:0048508)	2.66	1.08E-02
homologous chromosome segregation (GO:0045143)	2.66	1.08E-02
mitotic cell cycle phase transition (GO:0044772)	2.66	2.34E-04
regulation of nuclear division (GO:0051783)	2.66	1.08E-02
embryonic morphogenesis (GO:0048598)	2.65	1.32E-03
chromosome organization involved in meiotic cell cycle (GO:0070192)	2.63	9.11E-04
maturation of LSU-rRNA (GO:0000470)	2.63	4.23E-02
mitotic cell cycle (GO:0000278)	2.62	2.95E-29
regulation of mitotic cell cycle (GO:0007346)	2.62	2.22E-07
chromosome organization (GO:0051276)	2.61	9.17E-24
anatomical structure arrangement (GO:0048532)	2.6	8.48E-07
cell cycle phase transition (GO:0044770)	2.6	4.35E-04
meristem structural organization (GO:0009933)	2.59	3.73E-05
mitotic cell cycle process (GO:1903047)	2.59	7.25E-22
nuclear division (GO:0000280)	2.57	2.15E-16
double-strand break repair (GO:0006302)	2.56	4.98E-14

meiotic chromosome segregation (GO:0045132)	2.56	1.14E-03
heterochromatin formation (GO:0031507)	2.54	5.56E-09
meiosis I (GO:0007127)	2.54	7.29E-07
meiosis I cell cycle process (GO:0061982)	2.54	5.02E-07
stomatal complex morphogenesis (GO:0010103)	2.52	3.55E-02
spindle assembly (GO:0051225)	2.5	1.38E-03
cytokinesis by cell plate formation (GO:0000911)	2.49	6.96E-06
reciprocal homologous recombination (GO:0140527)	2.49	8.05E-05
reciprocal meiotic recombination (GO:0007131)	2.49	8.05E-05
regulation of cell cycle (GO:0051726)	2.48	8.58E-23
stomatal complex development (GO:0010374)	2.46	1.75E-05
negative regulation of gene expression, epigenetic (GO:0045814)	2.44	6.65E-13
cell cycle (GO:0007049)	2.43	4.38E-48
post-transcriptional gene silencing (GO:0016441)	2.43	2.80E-02
cell cycle process (GO:0022402)	2.42	9.51E-43
DNA repair (GO:0006281)	2.42	2.71E-32
meiotic cell cycle (GO:0051321)	2.42	6.19E-16
regulation of transferase activity (GO:0051338)	2.42	1.28E-02
DNA recombination (GO:0006310)	2.4	6.92E-15
homologous recombination (GO:0035825)	2.4	1.08E-04
meiotic cell cycle process (GO:1903046)	2.4	2.56E-13
double-strand break repair via homologous recombination (GO:0000724)	2.39	6.41E-07
meiotic nuclear division (GO:0140013)	2.39	1.80E-08
recombinational repair (GO:0000725)	2.38	3.37E-07
regulation of DNA metabolic process (GO:0051052)	2.37	6.17E-08
DNA metabolic process (GO:0006259)	2.36	3.07E-42
organelle fission (GO:0048285)	2.36	1.04E-15
DNA damage response (GO:0006974)	2.34	7.89E-33
spindle organization (GO:0007051)	2.33	6.16E-04
epigenetic regulation of gene expression (GO:0040029)	2.31	3.28E-16
cytokinesis (GO:0000910)	2.29	4.42E-07
nucleotide-excision repair (GO:0006289)	2.26	4.69E-02
regulatory ncRNA processing (GO:0070918)	2.26	1.35E-02
ribosomal large subunit biogenesis (GO:0042273)	2.23	3.63E-04

plant epidermis morphogenesis (GO:0090626)	2.22	5.88E-06
microtubule-based process (GO:0007017)	2.21	1.06E-15
leaf morphogenesis (GO:0009965)	2.2	1.86E-05
xylem and phloem pattern formation (GO:0010051)	2.14	4.49E-03
trichome morphogenesis (GO:0010090)	2.13	1.49E-02
carpel development (GO:0048440)	2.12	4.97E-04
shoot system morphogenesis (GO:0010016)	2.12	2.62E-10
cell division (GO:0051301)	2.1	7.29E-12
male gamete generation (GO:0048232)	2.1	1.37E-02
microtubule cytoskeleton organization (GO:0000226)	2.1	1.64E-09
gamete generation (GO:0007276)	2.09	5.54E-03
gynoecium development (GO:0048467)	2.09	1.27E-04
chromatin remodeling (GO:0006338)	2.06	7.69E-16
regulatory ncRNA-mediated gene silencing (GO:0031047)	2.06	6.81E-06
regulation of organelle organization (GO:0033043)	2.05	1.63E-06
post-embryonic plant morphogenesis (GO:0090698)	2.04	7.16E-11
regulation of cell division (GO:0051302)	2.04	9.21E-03
chromatin organization (GO:0006325)	2.03	9.69E-16
regionalization (GO:0003002)	2.02	1.96E-07
maintenance of cell number (GO:0098727)	1.99	2.17E-02
stem cell population maintenance (GO:0019827)	1.99	2.17E-02
meristem development (GO:0048507)	1.97	1.05E-11
negative regulation of gene expression (GO:0010629)	1.97	1.41E-15
post-embryonic plant organ morphogenesis (GO:0090697)	1.97	1.62E-04
cell fate commitment (GO:0045165)	1.96	3.59E-02
trichome differentiation (GO:0010026)	1.96	3.59E-02
pattern specification process (GO:0007389)	1.94	4.12E-08
auxin transport (GO:0060918)	1.92	2.09E-02
hormone transport (GO:0009914)	1.91	1.66E-02
protein-DNA complex organization (GO:0071824)	1.91	5.60E-14
floral organ development (GO:0048437)	1.88	2.27E-09
floral whorl development (GO:0048438)	1.88	3.34E-07
cytoskeleton organization (GO:0007010)	1.85	4.07E-10
protein autophosphorylation (GO:0046777)	1.85	1.10E-04
RNA catabolic process (GO:0006401)	1.85	4.13E-02

response to brassinosteroid (GO:0009741)	1.84	4.88E-02
meristem maintenance (GO:0010073)	1.83	1.99E-05
androecium development (GO:0048466)	1.82	4.70E-02
flower development (GO:0009908)	1.82	4.97E-12
reproductive shoot system development (GO:0090567)	1.82	1.09E-12
stamen development (GO:0048443)	1.82	4.70E-02
anatomical structure formation involved in morphogenesis (GO:0048646)	1.81	4.91E-06
plant organ formation (GO:1905393)	1.8	4.62E-02
protein-RNA complex organization (GO:0071826)	1.8	5.77E-03
tissue development (GO:0009888)	1.79	3.20E-22
regulation of cellular component organization (GO:0051128)	1.78	9.84E-08
rRNA processing (GO:0006364)	1.78	2.76E-06
non-membrane-bounded organelle assembly (GO:0140694)	1.77	1.07E-02
post-embryonic plant organ development (GO:0090696)	1.77	2.85E-04
plant epidermis development (GO:0090558)	1.76	2.40E-07
plant organ morphogenesis (GO:1905392)	1.76	2.08E-13
protein-RNA complex assembly (GO:0022618)	1.76	2.67E-02
rRNA metabolic process (GO:0016072)	1.75	1.50E-06
anatomical structure morphogenesis (GO:0009653)	1.73	4.39E-28
negative regulation of macromolecule metabolic process (GO:0010605)	1.73	3.47E-16
negative regulation of macromolecule biosynthetic process (GO:0010558)	1.72	4.96E-14
ribonucleoprotein complex biogenesis (GO:0022613)	1.72	3.40E-10
cell wall polysaccharide metabolic process (GO:0010383)	1.71	1.75E-02
negative regulation of biosynthetic process (GO:0009890)	1.7	1.41E-13
negative regulation of cellular biosynthetic process (GO:0031327)	1.7	1.28E-13
negative regulation of metabolic process (GO:0009892)	1.7	4.65E-16
regulation of developmental growth (GO:0048638)	1.7	3.77E-03
cell wall macromolecule metabolic process (GO:0044036)	1.68	9.87E-03
cellular developmental process (GO:0048869)	1.68	9.20E-17
multicellular organismal reproductive process (GO:0048609)	1.68	3.77E-03
negative regulation of cellular metabolic process (GO:0031324)	1.67	3.07E-13
ribosome biogenesis (GO:0042254)	1.67	1.10E-06

cell differentiation (GO:0030154)	1.66	1.49E-14
cell morphogenesis (GO:0000902)	1.66	1.37E-07
sexual reproduction (GO:0019953)	1.66	4.97E-06
supramolecular fiber organization (GO:0097435)	1.66	3.43E-02
embryo development (GO:0009790)	1.64	7.38E-12
growth (GO:0040007)	1.64	5.89E-09
negative regulation of cellular process (GO:0048523)	1.63	2.34E-18
regulation of hormone levels (GO:0010817)	1.63	2.76E-04
nucleic acid metabolic process (GO:0090304)	1.62	2.00E-41
organelle assembly (GO:0070925)	1.62	4.02E-02
phyllome development (GO:0048827)	1.62	7.06E-10
cell growth (GO:0016049)	1.61	1.24E-06
embryo development ending in seed dormancy (GO:0009793)	1.61	7.55E-10
shoot system development (GO:0048367)	1.59	4.49E-14
developmental growth (GO:0048589)	1.58	6.12E-06
unidimensional cell growth (GO:0009826)	1.58	6.31E-04
developmental growth involved in morphogenesis (GO:0060560)	1.57	1.81E-04
reproductive structure development (GO:0048608)	1.57	1.33E-22
reproductive system development (GO:0061458)	1.57	2.05E-22
protein phosphorylation (GO:0006468)	1.56	1.13E-07
cellular component organization or biogenesis (GO:0071840)	1.55	1.88E-53
fruit development (GO:0010154)	1.55	2.72E-12
post-embryonic development (GO:0009791)	1.55	1.75E-26
protein-containing complex organization (GO:0043933)	1.55	2.24E-13
cellular component organization (GO:0016043)	1.54	4.42E-42
developmental process involved in reproduction (GO:0003006)	1.54	1.97E-24
negative regulation of biological process (GO:0048519)	1.54	1.22E-17
plant organ development (GO:0099402)	1.54	6.20E-18
reproductive process (GO:0022414)	1.54	1.59E-30
root morphogenesis (GO:0010015)	1.54	2.78E-03
gametophyte development (GO:0048229)	1.53	2.88E-06
nucleobase-containing compound metabolic process (GO:0006139)	1.53	6.28E-38
regulation of developmental process (GO:0050793)	1.53	3.05E-12
seed development (GO:0048316)	1.53	8.77E-11
leaf development (GO:0048366)	1.51	2.47E-03

organelle organization (GO:0006996)	1.51	1.70E-19
regulation of biological quality (GO:0065008)	1.51	6.85E-06
peptidyl-amino acid modification (GO:0018193)	1.5	4.71E-02
RNA processing (GO:0006396)	1.5	1.82E-10
nucleic acid biosynthetic process (GO:0141187)	1.49	3.59E-14
polysaccharide metabolic process (GO:0005976)	1.49	4.95E-04
system development (GO:0048731)	1.49	7.31E-28
anatomical structure development (GO:0048856)	1.48	2.16E-43
cell wall organization or biogenesis (GO:0071554)	1.48	2.48E-05
cellular component biogenesis (GO:0044085)	1.48	1.58E-16
developmental process (GO:0032502)	1.48	7.72E-45
pollen development (GO:0009555)	1.48	3.37E-03
regulation of reproductive process (GO:2000241)	1.48	2.14E-02
root development (GO:0048364)	1.48	4.28E-06
root system development (GO:0022622)	1.48	3.32E-06
external encapsulating structure organization (GO:0045229)	1.47	1.35E-02
multicellular organism development (GO:0007275)	1.47	1.16E-35
RNA biosynthetic process (GO:0032774)	1.47	2.54E-12
multicellular organismal process (GO:0032501)	1.46	5.58E-38
cellular response to stress (GO:0033554)	1.44	5.23E-12
nucleobase-containing compound biosynthetic process (GO:0034654)	1.44	5.32E-14
cellular component assembly (GO:0022607)	1.43	5.20E-07
regulation of multicellular organismal process (GO:0051239)	1.43	1.52E-03
regulation of multicellular organismal development (GO:2000026)	1.41	4.07E-02
phosphorylation (GO:0016310)	1.4	5.48E-05
macromolecule metabolic process (GO:0043170)	1.39	6.73E-57
protein-containing complex assembly (GO:0065003)	1.39	1.27E-02
RNA metabolic process (GO:0016070)	1.39	7.62E-12
macromolecule biosynthetic process (GO:0009059)	1.37	3.49E-19
regulation of response to stimulus (GO:0048583)	1.36	8.93E-05
carbohydrate metabolic process (GO:0005975)	1.34	1.39E-04
gene expression (GO:0010467)	1.34	4.18E-12
protein localization (GO:0008104)	1.34	1.01E-02
cellular macromolecule localization (GO:0070727)	1.33	8.35E-03
primary metabolic process (GO:0044238)	1.33	2.69E-60

cellular biosynthetic process (GO:0044249)	1.32	2.70E-21
cellular response to stimulus (GO:0051716)	1.32	1.07E-15
response to stress (GO:0006950)	1.32	2.14E-22
cellular localization (GO:0051641)	1.31	9.44E-04
macromolecule localization (GO:0033036)	1.31	2.06E-03
cellular process (GO:0009987)	1.3	1.90E-93
biosynthetic process (GO:0009058)	1.28	2.81E-19
defense response to other organism (GO:0098542)	1.28	3.92E-02
macromolecule modification (GO:0043412)	1.28	2.44E-09
positive regulation of cellular process (GO:0048522)	1.28	3.21E-02
protein modification process (GO:0036211)	1.28	2.44E-07
regulation of biological process (GO:0050789)	1.27	6.62E-27
regulation of cellular process (GO:0050794)	1.27	5.09E-23
regulation of macromolecule metabolic process (GO:0060255)	1.27	3.44E-12
biological regulation (GO:0065007)	1.26	4.47E-28
metabolic process (GO:0008152)	1.26	6.21E-49
cell communication (GO:0007154)	1.25	6.46E-04
phosphate-containing compound metabolic process (GO:0006796)	1.25	7.52E-04
phosphorus metabolic process (GO:0006793)	1.25	8.37E-04
positive regulation of biological process (GO:0048518)	1.25	5.94E-03
regulation of macromolecule biosynthetic process (GO:0010556)	1.25	1.21E-08
regulation of metabolic process (GO:0019222)	1.25	1.04E-11
response to external stimulus (GO:0009605)	1.25	9.48E-04
cellular metabolic process (GO:0044237)	1.24	1.91E-21
establishment of localization (GO:0051234)	1.24	5.73E-07
localization (GO:0051179)	1.24	1.02E-07
regulation of cellular biosynthetic process (GO:0031326)	1.24	2.78E-08
regulation of cellular metabolic process (GO:0031323)	1.24	5.28E-09
regulation of gene expression (GO:0010468)	1.24	1.38E-07
response to abiotic stimulus (GO:0009628)	1.24	1.45E-06
response to stimulus (GO:0050896)	1.24	2.21E-23
signal transduction (GO:0007165)	1.24	3.14E-03
signaling (GO:0023052)	1.24	2.74E-03
regulation of biosynthetic process (GO:0009889)	1.23	4.47E-08
response to oxygen-containing compound (GO:1901700)	1.23	2.05E-03

protein metabolic process (GO:0019538)	1.22	3.31E-08
transport (GO:0006810)	1.21	1.52E-04
organonitrogen compound metabolic process (GO:1901564)	1.19	1.59E-08
regulation of primary metabolic process (GO:0080090)	1.18	2.17E-03
response to chemical (GO:0042221)	1.16	8.71E-03
GO Ontology database DOI: 10.5281/zenodo.12173881 Released 2024-06-17		

GO Ontology database DOI: 10.5281/zenodo.12173881 Released 2024-06-17

Table S2. 152 genes involved in GO terms related to flower development

**AGI code**	**Gene symbol**	**Description**
AT4G31160	DCAF1	DDB1- and CUL4-associated factor homolog 1;DCAF1;PTN000319759;orthologs
AT5G12330	LRP1	Protein LATERAL ROOT PRIMORDIUM 1;LRP1;PTN004844870;orthologs
AT5G21150	AGO9	Protein argonaute 9;AGO9;PTN000527500;orthologs
AT4G17300		Asparagine--tRNA ligase, chloroplastic_mitochondrial;SYNO;PTN000502488;orthologs
AT2G45650	**AGL6**	Agamous-like MADS-box protein AGL6;AGL6;PTN001378582;orthologs
AT3G53020	RPL24B	Large ribosomal subunit protein eL24y;RPL24B;PTN000082568;orthologs
AT1G65010		Putative WEB family protein At1g65010, chloroplastic;At1g65010;PTN000570347;orthologs
AT2G15790	CYP40	Peptidyl-prolyl cis-trans isomerase CYP40;CYP40;PTN000116556;orthologs
AT4G29860	DSE1	Protein DECREASED SIZE EXCLUSION LIMIT 1;DSE1;PTN002323447;orthologs
AT4G20910	**HEN1**	Small RNA 2'-O-methyltransferase;HEN1;PTN001468451;orthologs
AT3G63530	BB	E3 ubiquitin-protein ligase BIG BROTHER;BB;PTN000342048;orthologs
AT1G24260	**SEP3**	Developmental protein SEPALLATA 3;SEP3;PTN000235595;orthologs
AT4G28490	RLK5	Receptor-like protein kinase 5;RLK5;PTN000703197;orthologs
AT3G13890	MYB26	Transcription factor MYB26;MYB26;PTN000068067;orthologs
AT5G37020	ARF8	Auxin response factor 8;ARF8;PTN000775340;orthologs
AT3G04680	CLPS3	Protein CLP1 homolog;CLPS3;PTN000298146;orthologs
AT1G75080	BZR1	Protein BRASSINAZOLE-RESISTANT 1;BZR1;PTN000772431;orthologs
AT1G68480	**JAG**	Zinc finger protein JAGGED;JAG;PTN001237677;orthologs
AT1G19270	DA1	Protein DA1;DA1;PTN002383487;orthologs
AT4G20270	BAM3	Leucine-rich repeat receptor-like serine_threonine-protein kinase BAM3;BAM3;PTN000701525;orthologs
AT4G18570		AT4g18560_F28J12_220;At4g18570;PTN000575828;orthologs
AT4G30520		Probable LRR receptor-like serine_threonine-protein kinase At4g30520;At4g30520;PTN000701618;orthologs
AT5G14070	GRXC8	Glutaredoxin-C8;GRXC8;PTN000018792;orthologs
AT5G11320	YUC4	Probable indole-3-pyruvate monooxygenase YUCCA4;YUC4;PTN000545984;orthologs
AT3G54560	H2AV	Histone H2A variant 1;H2AV;PTN001518489;orthologs

AT5G05560	APC1	Anaphase-promoting complex subunit 1;APC1;PTN000302762;orthologs
AT5G06839	TGA10	Transcription factor TGA10;TGA10;PTN001119541;orthologs
AT1G69180	**CRC**	Protein CRABS CLAW;CRC;PTN004859579;orthologs
AT1G19850	ARF5	Auxin response factor 5;ARF5;PTN000774866;orthologs
AT1G09000	ANP1	Mitogen-activated protein kinase kinase kinase ANP1;ANP1;PTN001554428;orthologs
AT2G45190	YAB1	Axial regulator YABBY 1;YAB1;PTN000783201;orthologs
AT5G20240	**PI**	Floral homeotic protein PISTILLATA;PI;PTN000235739;orthologs
AT1G52150	ATHB-15	Homeobox-leucine zipper protein ATHB-15;ATHB-15;PTN000675715;orthologs
AT3G53040		Late embryogenesis abundant protein At3g53040;At3g53040;PTN000581998;orthologs
AT3G50330	HEC2	Transcription factor HEC2;HEC2;PTN001395437;orthologs
AT2G02970	APY6	Probable apyrase 6;APY6;PTN000207687;orthologs
AT4G36930	**SPT**	Transcription factor SPATULA;SPT;PTN001395447;orthologs
AT4G27330	**SPL**	Protein SPOROCYTELESS;SPL;PTN002121865;orthologs
AT5G27320	GID1C	Gibberellin receptor GID1C;GID1C;PTN000546399;orthologs
AT3G15510	NAC056	NAC transcription factor 56;NAC056;PTN000786231;orthologs
AT1G53160	SPL4	Squamosa promoter-binding-like protein 4;SPL4;PTN001261854;orthologs
AT1G30330	ARF6	Auxin response factor 6;ARF6;PTN001584072;orthologs
AT1G72520	LOX4	Lipoxygenase 4, chloroplastic;LOX4;PTN000206331;orthologs
AT3G57920	SPL15	Squamosa promoter-binding-like protein 15;SPL15;PTN001261850;orthologs
AT3G44600	CYP71	Peptidyl-prolyl cis-trans isomerase CYP71;CYP71;PTN000117364;orthologs
AT1G34640		At1g34640;At1g34640;PTN002212284;orthologs
AT5G05660	NFXL2	NF-X1-type zinc finger protein NFXL2;NFXL2;PTN002644125;orthologs
AT2G28610	WOX3	WUSCHEL-related homeobox 3;WOX3;PTN001215971;orthologs
AT2G42830	**AGL5/SHP2**	Agamous-like MADS-box protein AGL5;AGL5;PTN000235643;orthologs
AT5G02600	NAKR1	Protein SODIUM POTASSIUM ROOT DEFECTIVE 1;NAKR1;PTN000517413;orthologs
AT2G23140	PUB4	U-box domain-containing protein 4;PUB4;PTN002761907;orthologs
AT5G16780	DOT2	SART-1 family protein DOT2;DOT2;PTN000365785;orthologs
AT3G02130	RPK2	LRR receptor-like serine_threonine-protein kinase RPK2;RPK2;PTN001231827;orthologs
AT5G67440	NPY3	BTB_POZ domain-containing protein NPY3;NPY3;PTN000789590;orthologs

AT1G75520	SRS5	Protein SHI RELATED SEQUENCE 5;SRS5;PTN000787954;orthologs
AT4G26000	PEP	RNA-binding KH domain-containing protein PEPPER;PEP;PTN000032782;orthologs
AT1G26310	**CAL**	Transcription factor CAULIFLOWER;CAL;PTN000235767;orthologs
AT1G65620	AS2	Protein ASYMMETRIC LEAVES 2;AS2;PTN000769838;orthologs
AT5G23150	**HUA2**	ENHANCER OF AG-4 protein 2;HUA2;PTN000284818;orthologs
AT1G64520	RPN12A	26S proteasome non-ATPase regulatory subunit 8 homolog A;RPN12A;PTN001391081;orthologs
AT1G23290	RPL27AB	Large ribosomal subunit protein uL15y;RPL27AB;PTN001366144;orthologs
AT3G10910	ATL72	RING-H2 finger protein ATL72;ATL72;PTN002305218;orthologs
AT5G62000	ARF2	Auxin response factor 2;ARF2;PTN000775366;orthologs
AT4G15180	ATXR3	Histone-lysine N-methyltransferase ATXR3;ATXR3;PTN001874967;orthologs
AT3G57290	TIF3E1	Eukaryotic translation initiation factor 3 subunit E;TIF3E1;PTN000035188;orthologs
AT3G54610	GCN5;HAG1	Histone acetyltransferase GCN5;HAG1;PTN001485104;orthologs
AT2G33810	SPL3	Squamosa promoter-binding-like protein 3;SPL3;PTN001582023;orthologs
AT2G06990	**HEN2**	DExH-box ATP-dependent RNA helicase DExH10;HEN2;PTN002281681;orthologs
AT4G24670	TAR2	Tryptophan aminotransferase-related protein 2;TAR2;PTN001368252;orthologs
AT5G43270	SPL2	Squamosa promoter-binding-like protein 2;SPL2;PTN001261852;orthologs
AT2G28290	SYD	Chromatin structure-remodeling complex protein SYD;SYD;PTN000084891;orthologs
AT5G40350	MYB24	Transcription factor MYB24;MYB24;PTN000837284;orthologs
AT5G20490	XI-K	Myosin-17;XI-K;PTN000321436;orthologs
AT1G60800	NIK3	Protein NSP-INTERACTING KINASE 3;NIK3;PTN000703059;orthologs
AT1G72560	PSD	Exportin-T;PSD;PTN000409543;orthologs
AT4G36260	SRS2	Protein SHI RELATED SEQUENCE 2;SRS2;PTN000787956;orthologs
AT3G23130	**SUP**	Transcriptional regulator SUPERMAN;SUP;PTN001567600;orthologs
AT1G44970	PER9	Peroxidase 9;PER9;PTN000775424;orthologs
AT4G03090	NDX	Nodulin homeobox;NDX;PTN002182996;orthologs
AT3G49500	RDR6	RNA-dependent RNA polymerase 6;RDR6;PTN000555012;orthologs
AT1G73590	**PIN1**	Auxin efflux carrier component 1;PIN1;PTN000788145;orthologs

AT4G39400	BRI1	Protein BRASSINOSTEROID INSENSITIVE 1;BRI1;PTN001563781;orthologs
AT5G06100	MYB33	Transcription factor MYB33;MYB33;PTN000068101;orthologs
AT5G10310	EPFL1	EPIDERMAL PATTERNING FACTOR-like protein 1;EPFL1;PTN002104456;orthologs
AT1G17110	UBP15	Ubiquitin carboxyl-terminal hydrolase 15;UBP15;PTN000609035;orthologs
AT1G12140	FMOGS-OX5	Flavin-containing monooxygenase FMO GS-OX5;FMOGS-OX5;PTN001494208;orthologs
AT5G05690	CYP90A1	3beta,22alpha-dihydroxysteroid 3-dehydrogenase;CYP90A1;PTN000669441;orthologs
AT2G36910	ABCB1	ABC transporter B family member 1;ABCB1;PTN001542672;orthologs
AT2G31650	ATX1	Histone H3-lysine(4) N-trimethyltransferase ATX1;ATX1;PTN000350904;orthologs
AT1G01040	DCL1	Endoribonuclease Dicer homolog 1;DCL1;PTN000383802;orthologs
AT4G36920	**AP2**	Floral homeotic protein APETALA 2;AP2;PTN000795332;orthologs
AT1G34210	SERK2	Somatic embryogenesis receptor kinase 2;SERK2;PTN000702382;orthologs
AT1G13710	CYP78A5	Cytochrome P450 78A5;CYP78A5;PTN001210443;orthologs
AT5G42080	DRP1A	Phragmoplastin DRP1A;DRP1A;PTN000170425;orthologs
AT4G16340	SPIKE 1	Guanine nucleotide exchange factor SPIKE 1;SPK1;PTN000594308;orthologs
AT1G68130	IDD14	Protein indeterminate-domain 14;IDD14;PTN000149430;orthologs
AT5G09750	HEC3	Transcription factor HEC3;HEC3;PTN001395436;orthologs
AT3G58780	**AGL1/SHP1**	Agamous-like MADS-box protein AGL1;AGL1;PTN002631964;orthologs SHATTER PROOF1_SHP1
AT1G62360	**STM**	Homeobox protein SHOOT MERISTEMLESS;STM;PTN000220142;orthologs
AT2G03710	**AGL3/SEP4**	Agamous-like MADS-box protein AGL3;AGL3;PTN001378557;orthologs
AT4G37810	EPFL2	EPIDERMAL PATTERNING FACTOR-like protein 2;EPFL2;PTN002430263;orthologs
AT1G30490	ATHB-9	Homeobox-leucine zipper protein ATHB-9;ATHB-9;PTN000675714;orthologs
AT5G14010	**KNU**	Zinc finger protein KNUCKLES;KNU;PTN002402997;orthologs
AT2G34710	ATHB-14	Homeobox-leucine zipper protein ATHB-14;ATHB-14;PTN000675717;orthologs
AT4G14723	EPFL4	EPIDERMAL PATTERNING FACTOR-like protein 4;EPFL4;PTN002847545;orthologs

AT3G22780	TSO1	CRC domain-containing protein TSO1;TSO1;PTN000278387;orthologs
AT4G09960	**AGL11/STK**	Agamous-like MADS-box protein AGL11;AGL11;PTN002632071;orthologs
AT5G07180	ERL2	LRR receptor-like serine_threonine-protein kinase ERL2;ERL2;PTN000702884;orthologs
AT4G29010	AIM1	Peroxisomal fatty acid beta-oxidation multifunctional protein AIM1;AIM1;PTN000592883;orthologs
AT3G02310	**SEP2**	Developmental protein SEPALLATA 2;SEP2;PTN000235593;orthologs
AT1G59640	BPE	Transcription factor BPE;BPE;PTN000286126;orthologs
AT2G23950		Probable LRR receptor-like serine_threonine-protein kinase At2g23950;At2g23950;PTN001563433;orthologs
AT4G28530	NAC074	At4g28530;NAC074;PTN001272534;orthologs
AT5G46880	HDG5	Homeobox-leucine zipper protein HDG5;HDG5;PTN000675689;orthologs
AT3G07050	NSN1	Guanine nucleotide-binding protein-like NSN1;NSN1;PTN000120050;orthologs
AT1G69120	**AP1**	Floral homeotic protein APETALA 1;AP1;PTN000235770;orthologs
AT3G28860	ABCB19	ABC transporter B family member 19;ABCB19;PTN000657482;orthologs
AT5G61850	**LFY**	Protein LEAFY;LFY;PTN002188553;orthologs
AT3G49670	BAM2	Leucine-rich repeat receptor-like serine_threonine-protein kinase BAM2;BAM2;PTN000703273;orthologs
AT2G33860	ARF3	Auxin response factor 3;ARF3;PTN000774863;orthologs
AT5G23260	TT16	Protein TRANSPARENT TESTA 16;TT16;PTN001378611;orthologs
AT5G53400	BOB1	Protein BOBBER 1;BOB1;PTN000270348;orthologs
AT4G16270	PER40	Peroxidase 40;PER40;PTN001584129;orthologs
AT1G02065	SPL8	Squamosa promoter-binding-like protein 8;SPL8;PTN001582010;orthologs
AT2G01940	SGR5	Zinc finger protein SHOOT GRAVITROPISM 5;SGR5;PTN002439587;orthologs
AT2G41370	NPR5	Regulatory protein NPR5;NPR5;PTN001194162;orthologs
AT1G11130	SUB	Protein STRUBBELIG;SUB;PTN001563952;orthologs
AT2G02850	ARPN	Basic blue protein;ARPN;PTN002093328;orthologs
AT3G54340	**AP3**	Floral homeotic protein APETALA 3;AP3;PTN001378562;orthologs
AT2G01500	WOX6	WUSCHEL-related homeobox 6;WOX6;PTN001215967;orthologs
AT5G07280	**EMS1**	Leucine-rich repeat receptor protein kinase EMS1;EMS1;PTN000702398;orthologs
AT1G19180	TIFY10A	Protein TIFY 10A;TIFY10A;PTN002100996;orthologs

AT3G57670	WIP2	Zinc finger protein WIP2;WIP2;PTN002239072;orthologs
AT2G19520	MSI4	WD-40 repeat-containing protein MSI4;MSI4;PTN000522943;orthologs
AT4G33430	BAK1	BRASSINOSTEROID INSENSITIVE 1-associated receptor kinase 1;BAK1;PTN001563474;orthologs
AT1G08320	TGA9	Transcription factor TGA9;TGA9;PTN000774142;orthologs
AT4G04890	PDF2	Homeobox-leucine zipper protein PROTODERMAL FACTOR 2;PDF2;PTN000674419;orthologs
AT1G15330	PV42A	SNF1-related protein kinase regulatory subunit gamma-like PV42a;PV42A;PTN001417757;orthologs
AT1G05230	HDG2	Homeobox-leucine zipper protein HDG2;HDG2;PTN000674420;orthologs
AT5G45780		Probable LRR receptor-like serine_threonine-protein kinase At5g45780;At5g45780;PTN001563431;orthologs
AT5G17800	MYB56	Transcription factor MYB56;MYB56;PTN000068145;orthologs
AT2G42200	SPL9	Squamosa promoter-binding-like protein 9;SPL9;PTN001582022;orthologs
AT5G65700	BAM1	Leucine-rich repeat receptor-like serine_threonine-protein kinase BAM1;BAM1;PTN000703274;orthologs
AT1G79280	NUA	Nuclear-pore anchor;NUA;PTN001822594;orthologs
AT4G21530	APC4	Anaphase-promoting complex subunit 4;APC4;PTN000327450;orthologs
AT5G50570	SPL13B	Squamosa promoter-binding-like protein 13B;SPL13B;PTN001261856;orthologs
AT4G18960	**AG**	Floral homeotic protein AGAMOUS;AG;PTN000235646;orthologs
AT2G31160	LSH3	Protein LIGHT-DEPENDENT SHORT HYPOCOTYLS 3;LSH3;PTN000771489;orthologs
AT2G16910	**AMS**	Transcription factor ABORTED MICROSPORES;AMS;PTN000548734;orthologs
AT5G62230	ERL1	LRR receptor-like serine_threonine-protein kinase ERL1;ERL1;PTN000702886;orthologs
AT5G24330	ATXR6	Histone-lysine N-methyltransferase ATXR6;ATXR6;PTN002239308;orthologs
AT1G01510	AN	C-terminal binding protein AN;AN;PTN000107792;orthologs

Bold letter genes are involved in floral organ development.

TableS3. GO analysis of upregulated genes during flower development (1,080 genes)

**GO biological process complete**	**Fold enrichment**	**P-value**
proton export across plasma membrane (GO:0120029)	18.61	8.62E-03
meiosis I (GO:0007127)	7.14	2.13E-05
meiosis I cell cycle process (GO:0061982)	6.95	3.08E-05
reciprocal homologous recombination (GO:0140527)	6.83	1.40E-03
reciprocal meiotic recombination (GO:0007131)	6.83	1.40E-03
homologous recombination (GO:0035825)	6.2	3.79E-03
meiotic nuclear division (GO:0140013)	5.73	5.11E-05
nuclear division (GO:0000280)	4.84	4.14E-05
DNA recombination (GO:0006310)	4.21	4.06E-04
organelle fission (GO:0048285)	4.05	3.71E-04
meiotic cell cycle (GO:0051321)	3.9	2.72E-03
meiotic cell cycle process (GO:1903046)	3.89	1.09E-02
chromosome organization (GO:0051276)	3.42	1.77E-02
anatomical structure formation involved in morphogenesis (GO:0048646)	3.34	7.91E-03
cell cycle (GO:0007049)	3.21	1.91E-07
mitotic cell cycle (GO:0000278)	3.17	1.75E-02
monoatomic ion homeostasis (GO:0050801)	3.06	1.10E-02
intracellular chemical homeostasis (GO:0055082)	3.04	1.95E-02
cell cycle process (GO:0022402)	3.02	2.21E-05
monoatomic cation homeostasis (GO:0055080)	2.98	4.37E-02
floral organ development (GO:0048437)	2.9	4.14E-02
external encapsulating structure organization (GO:0045229)	2.69	1.60E-02
chemical homeostasis (GO:0048878)	2.58	1.04E-02
DNA metabolic process (GO:0006259)	2.5	9.21E-03
homeostatic process (GO:0042592)	2.37	4.46E-02
anatomical structure morphogenesis (GO:0009653)	1.92	3.43E-02
reproductive process (GO:0022414)	1.8	2.99E-04
transport (GO:0006810)	1.65	2.87E-03
establishment of localization (GO:0051234)	1.61	5.49E-03
localization (GO:0051179)	1.58	6.70E-03
cellular process (GO:0009987)	1.28	3.03E-06

GO Ontology database DOI: 10.5281/zenodo.12173881 Released 2024-06-17

TableS4. GO analysis of down-regulated genes during flower development (1,176 genes)

**GO biological process complete**	**Fold Enrichment**	**P-value**
cell cycle (GO:0007049)	2.63	2.11E-04
cell cycle process (GO:0022402)	2.6	1.34E-03
tissue development (GO:0009888)	2.2	1.98E-03
regulation of gene expression (GO:0010468)	1.52	9.34E-03
regulation of macromolecule biosynthetic process (GO:0010556)	1.51	1.11E-02
regulation of cellular biosynthetic process (GO:0031326)	1.48	3.35E-02
regulation of biosynthetic process (GO:0009889)	1.47	4.10E-02
developmental process (GO:0032502)	1.42	4.92E-02
regulation of biological process (GO:0050789)	1.41	2.61E-05
biological regulation (GO:0065007)	1.39	5.52E-05
regulation of cellular process (GO:0050794)	1.37	5.87E-03
cellular process (GO:0009987)	1.21	2.65E-03

GO Ontology database DOI: 10.5281/zenodo.12173881 Released 2024-06-17

Table S5. Oligonucleotide primers used for RT-PCR

Genes	Primer sequence (5' to 3')	Annealing temperature
HrAP2	TTCAGTTTGGAGGACTATGAGGATG	55°C
	ATCTACCGCATTTGTGAAGGGT	
HrGLO1	GAAAGAATGCTGGAAGAGGA	55°C
	GAGCAAACTAGTAGAGAATA	
HrGLO2	GGCTTACATATCTATTGCACC	55°C
	GTAGCGGTCACCAAGCAGTTA	
HrDEF-C1	ATGGGGAGGGGAAAGATAGAG	58°C
	TCAAGCGAGGCGGAGATCATG	
HrDEF-C3	CCAACAAGAGCTTAATCCGTGAG	55°C
	AGTCCCATTTCCGAGTGCAA	
HrDEF-C4	GAACCATAATTTGAGGCGGGAG	58°C
	ATCTGATGAGGAGGGCCG	
HrAG2	ATGATGAGCATGCTGGAGGCC	55°C
	TTAACCCGAACGGAGAGCAAGATG	
HrAG3	ATGATGAGCATGCTGGAGGCC	55°C
	TTAACCCGAACGGAGAGCAAGATG	
HrAGL6-C1	GTTGAGCTGAAGAGGATAGAGAAC	58°C
	TTACAGCATCCATCCAAGCATGAAG	
HrAGL6-C2	ATGGGAAGAGGCAGAGTAGAGC	55°C
	TCAAACAGCCCATCCTGGCATG	
HrAGL6-C3	ATGGGGAGGGGCCGAGTC	55°C
	TCAAAGAGCCCATCCCTGCTTGTAG	

Table S6. Accession numbers for each dataset

Library Name	Accession number
Habenaria radiata 3mm flower bud_1	DRR624927
Habenaria radiata 3mm flower bud_2	DRR624928
Habenaria radiata 3mm flower bud_3	DRR624929
Habenaria radiata 4mm flower bud_1	DRR624930
Habenaria radiata 4mm flower bud_2	DRR624931
Habenaria radiata 4mm flower bud_3	DRR624932
Habenaria radiata 5mm flower bud_1	DRR624933
Habenaria radiata 5mm flower bud_2	DRR624934
Habenaria radiata 5mm flower bud_3	DRR624935
Habenaria radiata leaf_1	DRR624936
Habenaria radiata leaf_2	DRR624937
Habenaria radiata leaf_3	DRR624938
